# Sexual risk behaviors among youth heads of household in Gikongoro, south province of Rwanda

**DOI:** 10.1186/1471-2458-12-225

**Published:** 2012-03-22

**Authors:** Joseph Ntaganira, Laura J Hass, Sheila Hosner, Lisanne Brown, Nancy B Mock

**Affiliations:** 1 Department of Epidemiology and Biostatistics, National University of Rwanda, School of Public Health, B.P. 5229 Kicukiro, Kigali, Rwanda; 2 Tulane University, Rwanda Country Office, No. 22 Avenue des Grands Lacs, B.P. 5266 Kiyovu, Kigali, Rwanda; 3 Department of International Health and Development, Tulane University School of Public Health and Tropical Medicine, 1440 Canal Street, Suite 2200, New Orleans, LA 70112, USA

**Keywords:** Youth Heads of Household, HIV/AIDS, Sexual risk behaviors, Gender differences, Rwanda

## Abstract

**Background:**

As a result of the 1994 genocide and AIDS, Rwanda has a crisis of orphans. In 2005, the Ministry of Local Governance and Social Affairs of Rwanda has reported one million vulnerable children. Many of these are not only orphans but also youth heads of households (YHH). The purpose of this study was to: (a) identify risk behaviors that expose YHH to HIV infection, (b) determine gender-specific high risk profiles and, (c) determine predictors of sexual onset.

**Methods:**

A household survey was conducted among 692 YHH, aged 12-24, all beneficiaries of a World Vision basic needs program in Gikongoro, Rwanda, from January to March 2004. Participants were interviewed using a structured questionnaire. Data was collected on socio-demographic variables, HIV/AIDS prevention knowledge and sexual risk behaviors. Bivariate analyses of the study variables were performed to examine differences between males and females. A logistic regression analysis was conducted to analyze factors that were independently associated with the debut of having sex.

**Results:**

Forty-one percent of respondents reported sexual onset before age 15. Males were more likely to start earlier than females (50.4% versus 26.7%) but females reported more sexual onset with an older partner. Fifty-eight percent of females had their first intercourse with a partner who was four or more years older than themselves. While sexual activity was low (1.75 mean lifetime sexual partner, 0.45 mean sexual partner last twelve months), sexual experience was related to less social connectedness and use of drugs. Having a close friend also appeared to be protective for sexual debut. The analysis also found that although YHH were aware of some prevention measures against HIV/AIDS, there was low (19.8%) knowledge of the "ABC" prevention program promoted by the government. In addition, despite 85% of respondents knowing someone who had died of AIDS, only 31% perceived themselves at risk of HIV infection, and there was very low (13.2%) condom use among the sexually experienced.

**Conclusions:**

Results suggest the urgent need of HIV prevention programs tailored to YHH that provide knowledge, enhance negotiations skills, and increase the perception of HIV infection risk among YHH in Rwanda.

## Background

Recent evidence suggests that the burden of new HIV infections is concentrated in developing countries [1]. Of the estimated 33.3 million people living with HIV/AIDS worldwide in 2010, almost 68% are in sub-Saharan Africa (SSA), with 2 million being children younger than 15 years of age [1]. AIDS is the leading cause of death among individuals aged 15-59, and as a consequence, an estimated 12 million children ages 0-17 have lost one or both parents to AIDS [2]. The orphan crisis in SSA is expected to increase dramatically within the coming years. By 2010, it was estimated that approximately 35 million children in SSA would have lost at least one parent to AIDS [2,3].

Youth in SSA continue to be one of the populations at greatest risk for HIV infection, particularly young women [4,5]. Evidence suggests that adolescents who initiate sexual activity early engage in behaviors that place them at high risk for negative health outcomes. Early age of sexual onset has been associated with an increased number of lifetime sexual partners and higher risk of unintended pregnancy [6], lowered intentions to use condoms in subsequent relationships [7], and increased likelihood of acquiring HIV [5]. Data on Rwandan youth suggest that small proportions of both females and males are sexually experienced at an early age. In the 2005 RDHS (Rwanda Demographic and Health Survey), only 5.2% of youth aged 10-19 reported having had sexual intercourse before the age of 15 years. However, by age 20-24, nearly 20% of women and 20.2% of men reported having sexual intercourse before age 18. Several factors appeared to be significantly interrelated: early sexual activity, nightclub attendance, cigarette smoking, drug use, and alcohol use. In Zambia, for example, adolescents who used alcohol are almost two times more likely to have had sex [8]. In Kenya, the relationship between alcohol and sexual initiation appears to be stronger for females than males, suggesting that substance use may be more deviant for females, and thus its association with sexual behavior may be stronger than for males [9].

Worldwide, adolescents are at a vulnerable age for compromising their health by engaging in behaviors that predispose them to an increased risk of HIV infection. Among this group, orphans, particularly those who are YHH, are proved to be the most vulnerable [2].

Several factors have been suggested as predictors for the adoption of high risk behavior by adolescents. Poverty, unemployment, and low levels of education appear to be linked to higher levels of adolescent sexual activity and less knowledge about HIV and AIDS [10,11]. Among young women, low wealth is particularly associated with earlier sexual debut, multiple sexual partners, and lowered condom use [12,13]. With regards to orphans, as their households are more likely to be poor, they become more vulnerable to transactional sex [10]. They may be driven into sex to ensure their survival, receive material goods to relieve poverty, or for protection; and this behavior is likely to be associated with increased HIV risk [10,11]. In Malawi, for example, concerns were expressed about female orphans' spontaneous reporting of exchanging sex for money or goods [14]. Transactional sex is particularly common in African street children among whom orphans may represent the majority [15]. Studies in South Africa and Nigeria found that half of the street children have engaged in sex in exchange for money, goods or protection [16,17].

In addition to increased chances of transactional sex, low socio-economic status appears to be associated with women having experienced physically forced sex [11,18-20]. Sexual abuse is particularly of significance for orphans as studies have shown that forced and coercive sex is a major route for sexually transmitted infections, HIV, and pregnancy [21,22]. Evidence from studies around the world shows that sexual abuse is frequently associated with a wide range of adverse health consequences including sexual dysfunction, low self-esteem, alcohol and drug use, depression, suicide attempts, and sexual risk taking [23,24]. High levels of psychosocial distress were also found, with females reporting more depressive symptoms and social isolation [25]. A recent study in Zimbabwe found that orphan status was associated with early onset of sexual activity and more pronounced psychosocial disorders [26].

Characteristics of the family and household such as parents' type of marriage, family structure, family stability, and living away from home are important factors associated with adolescent behaviors [18]. Research relevant to the situation of orphans shows that youth who live with both parents appear to be less likely to engage in sex than those who live with only one parent or someone other than their biological parent [8,19,20]. Living in a family with both parents present implies the availability of support, supervision, and behavioral control in the lives of adolescents [21].

School attendance has been widely shown to be a protective factor against a wide range of negative youth outcomes in SSA, particularly among females [27,28]. In South Africa for example, a study conducted among rural youth aged 14-24 years showed that school attendance was associated with lower-risk sexual behaviors and, among men, lower HIV prevalence [22]. In Zambia, current school attendance was significantly associated with both lower levels of sexual activity and consistent use of condoms, while in Ghana it was strongly related to delay of sexual onset [8,29]. However, orphans, particularly females, are at high risk for being out of school, as they are more likely to have the responsibility of taking care of the household. Studies from Rwanda conducted among orphan heads of households show that the proportion of YHH was significantly higher for females than for males [30].

While there is a wealth of research on risk behaviors among orphans, currently, not one study has been conducted with Rwandan YHH that systematically investigates the factors that could potentially contribute to their risk of contracting HIV. Rwanda is also in the process of recovering from the 1994 genocide where more than 800,000 people died. The combined effects of AIDS and the genocide have created a crisis of orphans in Rwanda, further enhanced by the widespread level of poverty. In 2005, according to UNICEF, an estimated 290,000 children under age 18 were double orphans while the Ministry of Local Governance and Social Affairs of Rwanda was reporting one million vulnerable children [2,31]. The purposes of this study were to: (a) identify behaviors that put YHH at risk for HIV infection, (b) determine gender-specific high risk profiles, and (c) determine predictors of such risk behaviors. Understanding how socio-demographic factors such as age, gender, and socioeconomic status confer vulnerability to unsafe sexual behaviors among youth living on their own is important for designing appropriate policies and programs. The issue is especially pertinent in Rwanda because of the level of poverty and the combined effects of HIV prevalence and genocide.

## Methods

A baseline, cross-sectional survey was conducted among YHH in Gikongoro district. Gikongoro is a remote, rural area located in southern Rwanda, one of the most densely populated and poorest countries in Africa. The study was carried out during the first quarter of 2004.

### Sample

A total of 692 male and female heads of households, ages 12-24 years, were included in the study. These YHH were identified by World Vision Rwanda, an international nongovernmental organization working in Gikongoro from a list of 886 youth, all beneficiaries of World Vision's basic needs program. YHH were selected based on criteria established by the community, before the implementation of a new project aiming at providing psychosocial support to YHH. The age and health status of the caretaker were the primary indicators in assessments of vulnerability; however, other factors such as number of persons in the household and food insecurity were also considered important. In Rwanda, an orphan is defined as a child who has lost one or both parents. In this study, in accordance with the WHO, a "youth" head of household is defined inclusively as being a young person aged below 25 years who is in charge of the household.

### Instrument development

The questionnaire was developed through a multiphase process to ensure its appropriateness both culturally and linguistically. Local concepts of psychosocial well-being and distress were identified through four, gender-mixed focus groups with 32 youth, conducted in the local language, Kinyarwanda. Two of the focus groups were with adolescents aged 15-19 years and two were with young adults aged 20-25 years. Discussions were recorded, transcribed verbatim, translated into English, and analyzed thematically. Information was also gathered from 30 street children, ages 13-17, who participated in a free listing activity [32]. These children were living in Butare, at approximatively 15 kilometres from Gikongoro. Researchers used these findings to select items from the Center for Epidemiological Studies Depression (CES-D) survey instrument to be used in the questionnaire. The draft questionnaire was then translated into Kinyarwanda before being submitted to a technical committee of experts for review. These experts included professors from Tulane University and local professionals including National University of Rwanda professors, NGO and governmental representatives, and persons with psychosocial and mental health expertise. Finally, the questionnaire was refined after pre-testing and piloting with 30 street children from Butare and 89 YHH from rural Kigali. None of the participants in the focus group discussions, free listing activity, or pre-testing of questionnaire were included in the final data collection.

### Study procedures

Twenty trained data collectors of both gender conducted the fieldwork under the supervision of the National University of Rwanda, School of Public Health. Face-to-face gender-matched interviews, lasting 1-2 hours, took place in the homes of respondents to ensure confidentiality and improve disclosure. Informed consent was sought prior to each interview and each respondent was informed of his or her right to withdraw from the study at any time without penalty. The consent statement was read to the potential respondents. They were informed that the research should not put them at risk. However, each respondent was informed that some questions may concern topics that are difficult or upsetting and that they were free to end the interview at any time.

Data collectors were sensitized to anticipate adverse consequences among respondents and trained to pause and offer support if interviews provoked a strong emotional reaction, continuing only when the respondent agreed. The discovery of youth in danger, such as suicidal ideation or a history of physical or sexual abuse and neglect, were automatically reported to World Vision staff for referral and further intervention.

A small bag of household items consisting of a candle, matches, soap, rice, and flour, was provided as a token of appreciation and in compensation for participants' time. Ethical approval for the study was obtained from the Tulane University Health Sciences Institutional Review Board and the Rwandan Ethical Review Board under the Ministry of Health.

### Measures

#### Demographic characteristics

Demographic characteristics of YHH included were age, gender, number of years as head of household, number of other children and youth in the household, current school enrolment, education achievement, and household characteristics (assets owned, socioeconomic status index).

#### Knowledge of HIV/AIDS prevention and perception of HIV/AIDS infection risk

Six items were used to measure HIV/AIDS prevention knowledge including "abstain from sex", "being faithful to one partner", "condom use", "avoid injections", "avoid sharing razors/blades", and "avoid traditional doctors". Only "yes"/"no" answers were possible. ABC knowledge of HIV prevention was measured by three factors: abstinence, being faithful to one partner and condom use. A respondent was considered as having correct knowledge of HIV prevention when he/she responded "yes" to the three questions. Respondents were also asked their perception of HIV infection risk, if they knew someone who had died of AIDS and their relationship to that person, and if they knew of a place where they could obtain a condom.

#### Sexual activity

Respondents were asked to describe their relationship with their current partner, if applicable. Sexual intercourse was defined as full penile-vaginal penetration. Those who reported a history of sexual intercourse responded to further questions, including the age of first sexual intercourse, age of first partner, condom use at the first sexual encounter, number of lifetime sexual partners, and partners in the last twelve months. For those who were sexually active in the previous twelve months, information was gathered regarding the use of condoms at the last sexual encounter. Sexually active female youth were asked if they have ever been pregnant, sexually active male youth, if they have ever gotten a girl pregnant. Transactional sex was assessed by asking respondents whether they had ever had sex expecting someone to take care of them or their siblings, or received anything in exchange for sex, such as money or gifts.

#### Other risk behaviors: alcohol consumption, use of drugs

Youth were asked whether they had ever used alcohol and/or marijuana. For those who used alcohol, the frequency of their consumption was collected.

#### Community connectedness and depression

Social connectedness to the community was measured by four factors: community group affiliation, peer relationships, having moved from the area more than twice in the last five years, and perceived social support [33]. The assumption being that youth who do not belong to a community group or who have moved often were less likely to feel socially connected. A scale was also generated to assess the level of adult support available to the youth. The scale consisted of four items including whether they had an adult in their life that they can always depend on, someone to give them advice and guidance, someone to comfort them when they are sad or sick, and an adult who would go to the authorities with them if they needed help. Depression was measured by using the 20-item standardized CES-D. Cronbach's alpha was calculated for each of these scales to assess internal consistency; scores were 0.87 and 0.84 respectively.

### Data analysis

Data were analyzed using SPSS 15.0 for Windows. Factor Analysis using Principal Components with varimax rotation was done. For this study, using the eigenvalue greater than 1.0 test, a minimum of 0.40 for factor loadings and 0.60 for alpha levels was established [34]. Cronbach's alpha was calculated to assess the internal consistency of scale items as reported above (adult support and depression). Socio-demographic, HIV/AIDS knowledge and sexual risk behavior variables were stratified by gender. Means and standard deviations were computed for continuous variables and proportions for categorical variables. Bivariate analyses of all the study variables were performed to examine differences between males and females heads of households. Chi-square analyses and its corresponding odds ratio, and 95% confidence interval were used to examine whether the bivariate association between pairs of dichotomous independent and dependent variables was significant and the magnitude of the association. Student's *t*-tests were computed to test the difference in means. A logistic regression analysis was used to assess the independent contribution of each factor in predicting the debut of having sex, after controlling for demographic variables and other independent variables. Only non-demographic variables that were significant in the bivariate analysis at *p *values less than 0.05 were entered into the model. Finally, a possible interaction with the outcome of interest was tested with the main effects.

## Results

### Respondents' socio-demographic characteristics

Socio-demographic characteristics of the 692 youth interviewed are presented in Table 1. There were slightly more male (53.6%) than female (46.4%) heads of households, with the majority (62.4%) aged 19-24, and 3.6% below age 15. The mean age for both female and male respondents was 20.1 years old (data not shown). Nearly 12% reported heading their household for ten or more years, while 46.3% became heads of household in the four years preceding the survey. Half reported taking care of 1-2 other children and 5% reported caring for 5 or more children. Nearly 20% reported living alone. The gross majority (95.9%) of YHH reported that one or both of their parents was dead; 0.9% indicated that their parents were alive and 3.2% were uncertain about the whereabouts of both parents. Eighty-seven percent of youth reported having attended school at some point, but only 6.2% achieved a level beyond primary and only 9.4% were currently enrolled in school.

**Table 1:** Socio-demographic characteristics of participants by gender

	Male *(n = 371)*		Female *(n = 321)*		Total *(n = 692)*	
	**n**	**%**	**n**	**%**	**n**	**%**

Age*						

12-14	13	3.5	12	3.7	25	3.6

15-18	110	29.6	125	38.9	235	34.0

19-24	248	66.8	184	57.3	432	62.4

Orphan status						

Double	270	72.8	214	66.7	484	69.9

Maternal	67	18.1	79	24.6	246	21.1

Paternal	18	4.9	16	5.0	34	4.9

Both alive	3	0.8	3	0.9	6	0.9

Uncertain	13	3.5	9	2.8	22	3.2

Number of years as head of household						

< 5	173	46.6	147	45.9	320	46.3

5-9	150	40.4	140	43.8	290	42.0

> = 10	48	12.9	33	10.3	81	11.7

Number of other children and adolescents in household***						

0	93	25.1	42	13.1	135	19.5

1-2	185	49.9	170	53.0	355	51.3

3-4	82	11.8	88	27.4	170	24.6

5 or more	11	3.0	21	6.5	32	4.6

School attendance						

Ever attended school	325	87.6	280	87.2	605	87.4

Currently in school	35	9.4	30	9.3	65	9.4

Education achievement						

Less than primary	285	77.0	233	72.8	518	75.1

Primary (7 years)	62	16.8	67	20.9	129	18.7

More than primary	23	6.2	20	6.3	43	6.2

Household characteristics						

3 or more rooms	272	74.3	252	79.2	524	76.6

Roof in tile or tin	340	91.6	298	92.8	638	92.2

Have a source of light	228	62.0	198	61,9	426	61.9

Latrine*	266	72.3	208	65.0	474	68.9

Eat one meal per day	161	43.4	140	43.6	301	43.4

Assets owned						

Farm/grazing land	351	94.6	307	95.6	658	95.1

Livestock	280	75.5	237	73.8	517	74.7

Mattress	35	9.4	39	12.1	74	10.7

Blanket	310	83.6	268	83.5	578	83.5

Shoes	222	59.8	207	64.5	429	62.0

Spare set of clothes**	259	69.8	252	78.5	511	73.8

Assets index (mean score, range 1-6)	3.5 (SD 1.47)		3.6 (SD 1.51)		3.6 (SD 1.50)	

Social connectedness						

Belong to 1 or more community groups	223	60.1	211	65.7	434	62.7

Peers relationships						

One close friend they can count on	340	91.6	293	91.3	633	91.5

Feel they belong to a group of friends	337	91.1	292	91.3	629	91.2

Moved 2 or more times during last 5 years**	124	33.7	69	21.6	193	28.1

Adult support (mean score, range 1-5)	3.3 (SD 1.06)		3.4 (SD 1.05)		3.4 (SD 1.06)	

Drink alcohol***	221	59.6	121	37.7	342	49.4

How often he/she drinks alcohol***^φ^						

Daily	23	10.4	2	1.7	25	7.3

Once a week	135	61.1	56	46.3	191	55.8

Once a month	50	22.6	51	42.1	101	29.5

Once a year/special event	13	5.9	12	9.9	25	7.3

Use drugs**	8	2.2	0	0.0	8	1.2

Depression index (mean score, range 1-55) ¶**	23.5 (SD 8.69)		25.2 (SD 9.26)		24.3 (SD 8.99)	

χ^2 ^*p ≤ 0.05; **p ≤ 0.01; ***p ≤ 0.001; ¶t-test **p ≤ 0.01

^φ^Only for those who drink alcohol (221 males, 121 females)

In terms of household characteristics and assets owned, males were more likely to reside in a house with a latrine than females (*p *< 0.05) but females were more likely to own a spare set of clothes (*p *< 0.05). Males were more likely to have moved more than two times in the five years preceding the survey (*p *< 0.05) and more likely to use alcohol and marijuana than females (*p *< 0.001 and *p *< 0.05 respectively). Although females were slightly more likely to report depressive symptoms than male heads of households, this difference was significant (*p *≤ 0.01).

### HIV/AIDS knowledge and perception of HIV risk infection

Table 2 presents knowledge of HIV/AIDS prevention measures and the perception of HIV risk infection by gender. All youth (99.9%) believe it is possible to avoid HIV infection. However, while 98.8% of respondents were aware that abstinence from sex was a prevention measure, only 57.9% reported use of condoms and 23.7% reported being faithful to one sexual partner as prevention measures. Knowledge of condom use as prevention against infection was significantly higher among males (*p *≤ 0.001). Knowledge of both condom use and being faithful to one sexual partner was reported by 19.8% of respondents (22.4% for males, 16.8% for females, *p *> 0.05). Knowledge of the full "ABC" message promoted by the National AIDS Control Program (Abstinence, Be faithful to one partner and Condom use) since 1995 was low at less than 20% of respondents and males were more likely to have higher scores than females (*p *≤ 0.001). Over half of respondents (56.9%) knew a place where to get condoms; however, males were significantly more likely than females to have that information.

**Table 2:** HIV/AIDS prevention knowledge and perception of HIV risk infection by gender

	Male *(n = 371)*		Female *(n = 321)*		Total *(n = 692)*			
	**n**	**%**	**n**	**%**	**n**	**%**	***χ*^2^**	***p***

Believe there are things a person can do to avoid HIV	370	99.7	321	100.0	691	99.9	0.86	1.000

HIV/AIDS knowledge	*n = 370*		*n = 321*		*n = 691*			

Abstain from sex	367	99.2	316	98.4	683	98.8	0.83	0.482

Use condom	243	65.7	157	48.9	400	57.9	19.81	**0.000**

Limit sex to one partner	95	25.7	69	21.5	164	23.7	1.65	0.210

Avoid injections	70	18.9	50	16.2	122	17.7	0.87	0.350

Avoid sharing razors	139	37.6	141	43.9	280	40.5	2.88	0.090

Avoid traditional doctors	119	32.2	122	38.0	241	34.9	2.58	0.108

"ABC" knowledge^φ^	83	22.4	54	16.8	137	19.8	17.24	**0.001**

Know anyone who had died or think has died of AIDS	311	83.8	277	86.3	588	85.0	0.81	0.394

Relationship to this person¶	*n = 311*		*n = 277*		*n = 588*		4.92	0.425

Parent	18	5.8	24	8.7	42	7.2		

Other family member	61	19.6	45	16.3	106	18.1		

Close friend	3	1.0	3	1.1	6	1.0		

Acquaintance	71	22.8	64	23.2	135	23.0		

Neighbor	158	50.9	140	50.7	298	50.7		

Perception of HIV risk	*n = 368*		*n = 319*		*n = 687*		2.78	0.425

No risk/possibility	251	68.2	222	69.6	473	68.9		

Small risk	40	10.9	26	8.2	66	9.6		

Moderate risk	68	18.5	58	18.2	126	18.3		

Great risk	9	2.4	13	4.1	22	3.2		

Know a place where one can get condoms	260	70.6	132	41.3	392	56.9	59.62	**0.000**

^φ ^Mentioned abstain from sex, use condom and limit sex to one partner

¶Calculated with the number of participants who know anyone who had died or think has died of AID as a denominator

A large majority of respondents (85%) knew someone who had died of AIDS, however, more than two thirds (68.9%) indicated they had no risk or possibility of contracting HIV and only 3.2% believed they had a high risk of HIV infection. Half of respondents (50.7%) reported knowing a neighbor who had died of AIDS.

### Sexual risk behaviors

When asked about their current romantic relationship, 58.4% of YHH reported that they had no boy/girlfriend (54.7% for males; 62.6% for females), 30.3% reported a steady sexual relationship with only one partner (32.1% for males; 28.3% for females), and 10.5% indicated having more than one sexual partner (12.9% for males; 7.8% for females). These differences according to gender are statistically significant (*p *< 0.05).

Table 3 displays the distribution of sexual risk behaviors by gender, the context of first experiences with sexual intercourse, and subsequent sexual experience. Thirty-three percent of respondents reported ever having had sex (n = 228), with males (36.9%) more likely to have had sex than females (28.3%). With regard to circumstances of first sex, differences between males and females YHH were found on a number of variables. YHH males reported a younger age of sexual onset with 29.2% of them having had sex under the age of 10 compared to only 11.1% for females. The mean age of first sex among those sexually experienced was 14 years for males and 16 years for females. Females were more likely to report older first sexual partners than males. Fifty-eight percent of females had their first intercourse with a partner who was four or more years older than themselves while 35% of male YHH first sex was with a younger partner. The mean age of the first partner was 21.80 years for females compared to 14.61 for males. Condom use during first intercourse was very low for both males and females (8.3%). In the age 15-19, almost 46% of respondents reported having had sex.

**Table 3:** Prevalence of Sexual Risk Behaviors by gender

	Male *(n = 371)*		Female *(n = 321)*		Total *(n = 692)*			
	**n**	**%**	**n**	**%**	**n**	**%**	***χ*^2^**	***p***

Ever had sex	137	36.9	91	28.3	228	32.9	5.732	**0.019**

Age of first sex							15.02	**0.001**

< 10	40	29.2	10	11.1	50	22.0		

10-14	29	21.2	14	15.6	43	18.9		

15-19	55	40.1	49	54.4	104	45.8		

> 19	13	9.5	17	18.9	30	13.2		

Mean age at first sex	14.06 (SD 4.19)		16.07 (SD 4.14)		14.85 (SD 4.28)		-3.54¶	**0.000**

Age of first partner							60.54	**0.000**

< 10	35	25.5	7	8.1	42	18.8		

10-14	33	24.1	6	7.0	39	17.5		

15-19	46	33.6	19	22.1	65	29.1		

20-24	19	13.9	24	27.9	43	19.3		

> 24	4	2.9	30	34.9	34	15.2		

Mean age of first partner	14.61 (SD 5.05)		21.80 (SD 7.92)		17.38 (SD 7.21)		-8.28¶	**0.000**

Age difference in first partner							80.57	**0.000**

Partner younger than respondent	48	35.0	2	2.3	50	22.4		

Partner same age as respondent	44	32.1	9	10.5	53	23.8		

Partner 1-3 yrs older	32	23.4	25	29.1	57	25.6		

Partner 4 or more yrs older	13	9.5	50	58.1	63	28.3		

Condom used at first sex	11	8.0	8	8.8	19	8.3	0.04	1.000

Ever had sex seeking for care	6	1.6	33	10.3	39	5.6	24.28	**0.000**

Ever received money or gift for sex	3	0.8	22	6.9	25	3.6	18.05	**0.000**

Ever given money or gifts for sex	22	5.9	0	0.0	22	3.2	19.66	**0.000**

Number sex partners in the past 12 months							7.99	**0.046**

None	94	68.6	59	64.8	153	67.1		

1	27	19.7	28	30.8	55	24.1		

2	9	6.6	4	4.4	13	5.7		

> = 3	7	5.1	0	0.0	7	3.1		

Mean number sex partners in the past 12 months	0.49 (SD 0.86)		0.40 (SD 0.57)		0.45 (SD 0.76)		0.91¶	0.363

Condom used at last sex	20	14.6	10	11.0	30	13.2	0.43	0.549

Reasons of not using a condom at last sex							25.85	**0.001**

No HIV risk	35	30.7	15	18.8	50	25.8		

Don't like them	6	5.3	6	7.5	12	6.2		

Partner did not want to use it	3	2.6	6	7.5	9	4.6		

Don't have them	16	14.0	8	10.0	24	12.4		

Want children	2	1.8	2	2.5	4	2.1		

Don't know condoms/AIDS	49	43.0	25	31.3	74	38.1		

Raped/Forced	1	0.9	13	16.3	14	7.2		

Other	2	1.8	5	6.3	7	3.6		

Number sex partners lifetime							4.64	0.200

1	73	53.3	60	67.4	133	58.8		

2	35	25.5	17	19.1	52	23.0		

3	16	11.7	6	6.7	22	9.7		

> = 4	13	9.5	6	6.7	19	8.4		

Mean number sex partners lifetime	1.88 (SD 1.34)		1.55 (SD 0.98)		1.75 (SD 1.22)		1.97¶	**0.049**

Ever been pregnant or gotten a girl pregnant	4	2.9	53	58.2	57	25	89.27	**0.000**

Age when she first got pregnant/he got a girl pregnant							0.81	0.666

< 15	0	0.0	1	2.0	1	1.9		

15-18	1	25.0	23	46.0	24	44.4		

> 18	3	75.0	26	52.0	29	53.7		

¶t value SD: Standard deviation

Although the numbers are very small, survival or transactional sex is higher among females than males. Ten percent of females, compared to only 1.6% of males reported having exchanged sex for protection. Almost 7% of females also indicated that they received money or gifts in exchange for sex. Conversely, only 5.9% of males reported ever having been given money or gifts for sex.

During the last twelve months, 67.1% of YHH reported being abstinent. A small number of respondents indicated more than one partner with 5.1% males reporting 3 partners or more. The mean number of sex partners in the last twelve months was equal among both gender groups (0.45 (SD 0.76)).

The proportion of condom use at last sexual intercourse was low for both males and females with more males (14.6%) using condoms compared to females (11%). Among males, the most common reason given for not using condoms was the perception that there was no risk of HIV infection (30.7%). Sixteen percent of females reported that they were forced to have sex or raped and 7.5% indicated that their partner did not want to use condoms. It is notable that 38.1% of those who did not use a condom during the last sexual intercourse declared that they didn't know a condom or AIDS.

Respondents reported few (1.75 average) lifetime sexual partners. Fifty-eight percent of females reported a history of pregnancy but only 2.9% of males reported getting a girl pregnant. Females reported 46% of pregnancies occurred between the ages of 15-18 and 52% over the age of 18.

### Correlates of sexual experience (Ever Had Sex)

The correlates of sexual experience in terms of socio-demographic characteristics, social behaviors, and the perception of HIV risk infection were analyzed (Table 4). Logistic regression results indicate that YHH under age 18 had a significantly lower adjusted odds ratio (AOR) of being sexually experienced (AOR = 0.60). YHH who indicated having a close friend they can count on were also less likely to report having had sex (AOR = 0.51). On the contrary, respondents who moved more than two times during the last five years, had a history of using marijuana, or perceived risk of HIV infection had a significantly higher likelihood of being sexually experienced (AOR = 1.85, 9.77, and 1.95, respectively).

**Table 4:** Bivariate analysis and logistic regression model predicting sexual onset among YHH in Gikongoro at the time of the survey

	Bivariate n = 692		Multivariate^φ^ n = 692	
	**Odds Ratio**	**95% CI**	**Odds Ratio**	**95% CI**

**Demographic characteristics**				

Age				

12-18	0.42***	0.28-0.62	0.60*	0.37-0.98

19-24	Ref.		Ref.	

Gender				

Male	1.48**	1.07-2.04	1.30	0.89-1.91

Female	Ref.		Ref.	

Orphan Status				

Double	Ref.		Ref.	

Maternal	0.85	0.57-1.25	0.73	0.46-1.15

Paternal	0.98	0.46-2.07	1.04	0.44-2.44

Both Alive	0.94	0.17-5.20	0.86	0.12-5.94

Uncertain	0.82	0.33-2.00	0.67	0.24-1.86

Number of years as head of household				

< 5	0.66**	0.48-0.91	0.78	0.54-1.15

≥ 5	Ref.		Ref.	

Number of other children & adolescents in household				

0	1.20	0.54-2.64	1.53	0.59-3.93

1-2	1.69	0.80-3.56	2.07	0.87-4.89

3-4	1.13	0.52-2.44	1.34	0.55-3.25

5 or more	Ref.		Ref.	

School attendance				

Ever attended school (Yes versus No)	1.63	0.97-2.74	N/A	

Currently in school (Yes versus No)	0.40**	0.20-0.76	0.51	0.25-1.05

Education completed				

None	1.18	0.61-2.28	N/A	

Primary (7 years)	0.81	0.39-1.68	N/A	

More than primary	Ref.			

**Household characteristics**				

Eat one meal per day	1.29	0.94-1.78	N/A	

Assets index (continuous)	0.91	0.82-1.01	N/A	

**Social connectedness**				

Belong to 1 or more community groups (Yes versus No)	0.84	0.61-1.17	N/A	

Peers relationship				

One close friend they can count on	0.55*	0.32-0.94	0.51*	0.28-0.94

Feel they belong to a group of friends	0.79	0.46-1.38	N/A	

Moved 2 or more times during last 5 years (Yes versus No)	1.89***	1.34-2.67	1.85**	1.22-2.79

Adult support (≤ 3 versus > 3)	1.11	0.79-1.54	N/A	

**Youth behaviors and emotional distress**				

Drink alcohol	1.31	0.95-1.80	N/A	

Use drugs	14.66**	1.79-119.9	9.77*	1.14-83.44

Depression (Cut-off = 24)	1.39*	1.01-1.91	1.14	0.78-1.65

**HIV/AIDS Prevention Knowledge**				

Abstain from sex	0.82	0.19-3.46	N/A	

Condom use	1.68**	1.21-2.34	1.22	0.83-1.79

Limit sex to one partner	0.99	0.69-1.45	N/A	

Avoid traditional doctors	1.07	0.77-1.49	N/A	

**Perception of HIV infection risk**				

No risk/possibility	Ref.		Ref.	

Small/Moderate/Great risk	2.19***	1.56-3.07	1.95***	1.32-2.86

*p ≤ 0.05; **p ≤ 0.01; ***p ≤ 0.001

^φ^Five basic socio-demographic variables (age group, gender, orphan status, number of years as HH, number of other children & adolescents in household) and variables that attained *P *< 0.01 in the bivariate analyses were entered into the logistic regression model. -N/A = Not Applicable; Ref. = Reference group; CI = Confidence Interval

Although not statistically significant in the logistic regressions, other important socio-demographic factors, i.e., gender male, years served as head of household, knowledge of condom as a prevention measure, and currently enrolled in school were associated with being sexually experienced in bivariate analyses. Respondents who were in school and heads of household for less than five years were less likely to be sexually active (OR = 0.40, OR = 0.66). Conversely, being a male had a significantly higher likelihood of being sexually experienced (OR = 1.48). Similarly, respondents with a higher depression score and those who were informed on condom use for HIV prevention were more likely to be sexually experienced (OR = 1.35, OR = 1.68).

## Discussion

This study provided the opportunity to look at HIV-related sexual risk behaviors among YHH aged 12-24, living on their own, in a rural area of Rwanda, Gikongoro district. The results show striking differences on many aspects with findings from the recent DHS in Rwanda with regard to youth and orphans [35].

Knowledge of the "ABC" HIV/AIDS messages is very low among YHH with only 19.8% of youth being able to cite all three prevention measures. Use of condom was particularly less likely to be known among females than males (48.9% versus 65.7%). Knowledge of both use of condom and being faithful to one sexual partner was also very low at less than 20%. Although 85% of YHH seem to know a person who died of AIDS, the majority (68.9%) do not perceive themselves at risk of HIV infection. Half of respondents (50.7%) reported knowing a neighbor who had died of AIDS, reflecting the high HIV prevalence in the community. Results from the 2005 RDHS show a similar trend of HIV knowledge although figures are much higher; 51% of young women and 54% of young men age 15-24 were shown to have a comprehensive knowledge (Use of condom and limiting sexual intercourse to one faithful uninfected partner) of the means of prevention and transmission of HIV/AIDS. In addition, 79.5% of women and 88.4% of men knew that the risk of contracting HIV/AIDS can be limited by using condoms.

Among YHH in Gikongoro, 36.9% of males and 28.3% of females reported a history of penetrative sexual intercourse. Respondents in this population reported a very young age of sexual debut with 41% before the age 15; males being more likely to start earlier than females (50.4% versus 26.7%). The 2005 RDHS again shows similar ratios between males and females, but much lower percentages of early sexual intercourse in the DHS study with more frequency among men (14%) than women (5%).

While sexual activity is higher among YHH respondents compared to youth of the same age in the general population, the use of condoms is not common. Thirty-three percent reported having had at least one partner during the past twelve months with only 13.2% using condoms during the last sexual intercourse. In the 12 months preceding the 2005 RDHS, approximately 5% of never-married women age 15-24 had had sexual intercourse. Among these women, 25% used a condom at their last sexual intercourse. Among never-married men age 15 to 24, approximately 9% reported having had sexual intercourse in the past 12 months and, among these, 39% used a condom at their last sexual intercourse. Similarly, the use of condoms appeared not common at first intercourse in this study population. On average, only 8.3% reported the use of condoms at first sex in our study compared to 12% for men and 7% for women among youth age 15-24 in the DHS.

Obviously, these findings suggest that YHH in Gikongoro might be sexually vulnerable and at high risk of HIV infection and poor health outcomes. While the results indicate that YHH are not very sexually active (1.75 mean lifetime sexual partner, 0.45 mean sex partner last twelve months) as in other SSA settings, research shows that early sexual onset is associated with lowered condom use in subsequent relationships [7], an increased risk of HIV infection [5], and higher likelihood of unintended pregnancy [6]. In fact, a sizable proportion of YHH respondents do not use condoms, had their first sexual intercourse at a very young age and with older partners, risk factors for HIV infection [27,36]. In addition, transactional sex, a factor that may limit their ability to negotiate the use of condoms [28], was reported by 10% of youth. Moreover, the lack of adult supervision and non condom use may have heightened risks of adverse reproductive health outcomes [37], as 58.2% of sexual experienced females reported a pregnancy.

Although there may be various circumstances explaining early onset of sexual activity among YHH in Rwanda, poverty may be an important factor vis-à-vis survival. However, in this study, no association emerged between assets, number of meals per day used as proxy for socio-economic status and sexual onset. This finding is inconsistent with evidence from other studies showing that low wealth and economic hardship may contribute to the engagement of youth in sexual activity [12,13]. It appears that the burden of HIV/AIDS and the loss of parents during the genocide impacts strongly on YHH. Poverty associated with lack of resources and the scarcity of land in an overpopulated country may increase youth's difficulty in meeting the needs of their siblings [38]; 43.4% of YHH reported having only one meal per day. For some youth, prostitution and survival sex may serve as a means of obtaining basic needs [14]. In fact, 7% of female respondents reported having received money or gifts for sex and 10.3% reported having sex in exchange for protection. Transactional sex coupled with the lack of adult support and the fact that a significant proportion of YHH reported their first sexual experience before the age 15 may also suggest possible exploitation and abuse.

This study has identified several factors related to sexual experience among YHH in Rwanda. Some of these are characteristics of youth themselves, while others are linked to the environment in which they are embedded. Having moved more than two times, marijuana use, and the perception of HIV risk infection were significantly associated with having ever had sex. In contrast, being in school and having a close friend were protective for sexual onset.

These findings are consistent with prior literature in indicating that sexual behaviors of youth are influenced by various factors. Research has highlighted the role of protective factors in explaining sexual behaviors among youth exposed to a high risk social environment [39]. For example, being well integrated within the community has beneficial effects across a range of health and social outcomes [34]. Respondents who had moved often and who did not have a close friend were thus unlikely to feel socially connected to the community. In addition, other studies in Rwanda have shown that YHH face stigma and marginalization [29] which could have a major impact on their behavior. The results of our study suggest that YHH may use sex as a means of gaining support and establishing themselves in the community. Belonging to community groups was also found to be protective, though the association was not significant.

Among the demographic factors, older age was, not surprisingly, associated with a higher likelihood of having had sex. In contrast, although male gender and years served as head of household were significantly associated to sexual onset at the bivariate level, the relationship does not persist in the final model. As in prior research, the findings showed that being out of school was associated with an elevated likelihood of being sexually experienced [29,40,41].

One noteworthy finding is with regards to the relationship between HIV/AIDS knowledge and behavior. Although the overall "ABC" knowledge in the sample was very low, a strong association was noticed at the bivariate level between condom use knowledge as a prevention measure and ever had sex. This finding is important as most youth programs providing information about HIV/AIDS focus essentially on abstinence-only education and not condom use. Despite the taboo surrounding the notion of sex, and because adolescents do not always see themselves at risk of HIV infection in spite of their high risk behavior, HIV prevention programs should go beyond abstinence and provide information and skills that will allow youth to make adequate choices, especially with regards to condom use [23,42].

The study findings concerning the perception of HIV risk infection by youth are also worthy of note. Only 31% of youth respondents perceived themselves at risk of HIV infection and they were more likely to have sexual intercourse. This low risk perception coupled with the incomplete HIV/AIDS prevention knowledge and the low level of condom use strongly suggest that HIV prevention campaigns throughout the country have failed in reaching and informing YHH about the risks of HIV infection, and indicate that these efforts should be reinforced. The low numbers of condom use in this sample of YHH did not allow for further analysis.

With regard to non-sexual behaviors and sexual initiation, the findings are consistent with prior research in SSA that shows a high likelihood of engaging in sex among drug users [43]. On the other hand, alcohol is often recognized as a prompt among youth toward adopting poor sexual behavior was not associated with the outcomes of interest [8,9]. Alcohol was found in over 50% of the respondents but it was consumed in moderation, mostly once a week. Similarly, despite the high level of depression observed in this population of YHH [25], psychosocial distress were not associated with early onset of sexual activity as reported by other studies in SSA [26].

Limitations of the study include the fact that the study methodology was cross-sectional for which the direction of causal relationships cannot be determined. In addition, measures of behavior such as sexual activity, number of partners, and age of sexual debut were based on self-report taken at the time of recruitment. Data are thus subject to reporting errors/bias and it is also possible that the use of face-to-face interviews to collect data may have further contributed to reporting errors. Because sexual behaviors are still taboo in Rwanda and may be a source of stigmatization, frequencies of behaviors identified in this study may therefore underestimate actual rates. Finally, this study was limited by sampling orphan YHH from a very remote area in Rwanda, recognized as the poorest in the country. Caution should therefore be exercised when generalizing the study findings to other demographic groups and geographic areas. More research is therefore needed to compare sexual risk behavior among orphans and non-orphans youth in urban or rural areas. A longitudinal study to examine the impact of parental illness or death on sexual behavior is also necessary to determine the effect and causal relationships between variables.

## Conclusions

This study reports on the prevalence of sexual risk behaviors and factors predicting sexual onset among YHH in Rwanda. The findings suggest that YHH have needs in terms of HIV/AIDS education and prevention interventions. Respondents demonstrated low knowledge levels of the "ABC" program, and early sexual activity with a higher proportion of older partners; condom use was not reported by the majority of respondents. These findings emphasize the importance of implementing HIV prevention programs that provide knowledge, enhance negotiations skills, target specific prevention-related social norms and increase the perception of HIV infection risk among youth. Life-skills education programs may represent an effective strategy in targeting potential protective measures and alleviating risk related factors.

## Competing interests

The authors declare that they have no competing interests.

## Authors' contributions

JN conceptualized the study, supervised data collection and participated in the interpretation of data and drafting of manuscript. LJH and SH participated in the interpretation of findings and drafting of manuscript. LB participated in study design and the interpretation of findings and drafting of manuscript. NBM participated in the interpretation of findings and drafting of manuscript. All the authors approved the manuscript.

## Pre-publication history

The pre-publication history for this paper can be accessed here:

http://www.biomedcentral.com/1471-2458/12/225/prepub
